# Methodological Considerations for Assessing Automatic Brightness Control in Endoscopy: Experimental Study

**DOI:** 10.3390/s23104932

**Published:** 2023-05-20

**Authors:** Nishitha Ravichandran, Preejith Sreelatha Premkumar, Mohanasankar Sivaprakasam

**Affiliations:** 1 Department of Electrical Engineering, Indian Institute of Technology, Madras, Chennai 600036, India; mohan@ee.iitm.ac.in; 2 Healthcare Technology Innovation Centre, Indian Institute of Technology, Madras, Chennai 600113, India; preejith@htic.iitm.ac.in

**Keywords:** automatic brightness control, image brightness, controller response, colour rendition

## Abstract

Endoscopy is a critical application that requires adaptable illumination to adjust to varying imaging conditions. Automatic brightness control (ABC) algorithms ensure optimal brightness throughout the image with rapid but smooth response and render the true colours of the biological tissue under examination. To achieve good image quality, high-quality ABC algorithms are necessary. In this study, we propose a three-assessment method approach for objectively evaluating ABC algorithms based on (1) image brightness and its homogeneity, (2) controller response and response time, and (3) colour rendition. We conducted an experimental study to assess the effectiveness of ABC algorithms in one commercial and two developmental endoscopy systems using the proposed methods. The results showed that the commercial system achieved good, homogeneous brightness within 0.4 s, and its damping ratio was 0.597, indicating a stable system, but its colour rendition was suboptimal. The developmental systems had control parameter values that resulted in either a sluggish response (over 1 s) or a fast (about 0.3 ms) but unstable response with damping ratios above 1, causing flickers. Our findings indicate that the interdependency among the proposed methods can establish tradeoffs in the overall ABC performance better than single-parameter approaches. The study establishes that comprehensive assessments using the proposed methods can contribute to designing new ABC algorithms and optimising already implemented ones for efficient performance in endoscopy systems.

## 1. Introduction

Endoscopy is a pervasive medical imaging process that enables the inspection of the interior cavities of the human body with a camera guided by an illumination source [1,2]. It is crucial to properly illuminate the region of interest (ROI) to obtain diagnostically correct and visually appealing images that aid in medical evaluation by emphasizing the characteristics of the observed scene [3,4,5,6,7]. During a live endoscopy examination, the relative movement among the endoscope, the subject, and the observed tissue causes reflectivity and absorption changes within the ROI [8]. The best-perceived image illumination can be maintained throughout the varying imaging conditions by altering light intensity, camera exposure, and gain [8,9,10]. Varying light intensities would help regulate the temperature at the endoscopy tip, avoiding injuries to the patients due to heat [11], but it is considered a coarse adjustment that would not be suitable for a live examination [8,9]. On the contrary, changing camera exposures results in a fine adjustment for a smooth viewing experience, but longer exposure times can cause motion blur [9]. Varying gain decreases the signal-to-noise ratio (SNR) [8] and leads to colour shifts if not applied properly to the image components [10]. The complex process of dynamically controlling the aforementioned parameters individually or in combination is addressed by the development of automatic brightness control (ABC) algorithms [5,12,13].

### 1.1. Related Work

There are three main concerns to ensuring the efficient performance of ABC algorithms. Firstly, the overall brightness of the ROI is needed not just to avoid over- or under-illumination but also to obtain a homogeneously illuminated image [12,14,15,16]. Secondly, the response of the control must be rapid and steady since endoscopy is a real-time process [13,17]. Finally, the true colours of the resultant images must be rendered for proper diagnosis [5,10,15]. To always ensure good images without causing any visual inconvenience during live endoscopy examination, the developed ABC algorithms need to be assessed based on all three concerns.

McCallum et al. conducted an experiment where eight experts were made to evaluate the image quality of hundred images with varying intensities acquired from endoscopic ear surgeries [11]. The subjective assessment gave feedback on anatomy, colour contrast, overall image quality, and suitability for operation but did not assess the characteristics of the control algorithm. Shreshtha et al. assess the image brightness and homogeneity of their developed algorithm for wireless capsule endoscopy (WCE) only, which gives no information about its response time and colour rendition [12]. Cavallotti et al. developed an FPGA-based system that tests the illumination of WCE for optimal performance consuming low power [17]. The system, however, gives no insights into image brightness and colour rendering. Sousa et al. [13] only assess an algorithm for a camera of a small form factor with an illumination source integrated into it to ensure optimal image brightness and a response time of less than 1 s. Thus, no work has addressed all three concerns specified for a comprehensive assessment of their ABC algorithms.

### 1.2. Proposed Work

From the literature, it is clear that there is no univocal opinion on objective assessment methodologies to assess the overall performance of the ABC algorithms of the endoscopy hardware or the control parameters used. Thus, this work proposes three assessment methodologies to assess (1) image brightness and the homogeneity of brightness, (2) the controller response and response time, and (3) colour rendition, and to establish an interdependency among them. The endoscopy image information is taken as the evaluation input for a blind assessment, making the methodologies suitable for evaluating the algorithms without the need for the endoscopy hardware. The assessments are aimed at providing insight into obtaining good image quality and an accurate representation of the observed scene by ensuring proper illumination rapidly and steadily without any colour shifts in the ROI. The assessments could also be part of regular quality checks of the endoscopy systems as the light source and camera hardware would deteriorate during its clinical lifetime, which could help in maintaining or improving image quality [18,19].

In Section 2, we provide a detailed description of the experimental setup, details of the ABC algorithms of endoscopy systems assessed by the three proposed assessment methods, and the emulations conducted to observe the performance of the algorithms. Section 3 outlines the key observations concerning the interdependency among the methods to provide comparative analyses of the algorithms. In Section 4, we discuss the inferences from the study and highlight the proposed methods. The final sections present the conclusions, limitations, and future directions for this research.

## 2. Methodology

**Figure 1:**
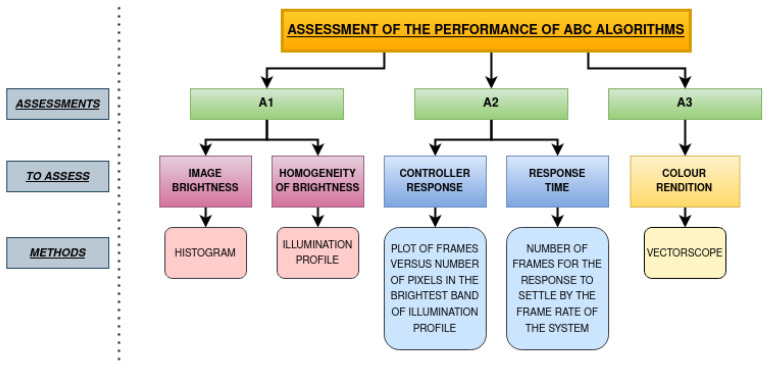
Representaion of the three methodological considerations to be made to assess the performance of the ABC algorithms.

The experimental evaluation of ABC algorithms was conducted in-house using a custom-built test setup and test charts. Three different endoscopy systems, including a commercially available system from a leading global manufacturer, were selected for assessment. Two in-house systems with custom ABC algorithms were also included for comparison. Each system’s controller response was recorded on video, and image frames were extracted for analysis. Three assessment methods (A1, A2, and A3) were used to evaluate ABC algorithm performance, as shown in Figure 1. Firstly, the image brightness was analysed from the histogram, and the homogeneity of the light intensity across the entire image was estimated using the illumination profile. Secondly, from the brightness information obtained from the A1, the controller response and its response time were calculated. Finally, the colour rendition was evaluated using vectorscope to observe the effect of illumination on rendering the true colours of the image. All three emulations were performed to gain further insight into the behaviour of each system’s ABC algorithm. The experimental setup and the experiments conducted are discussed elaborately in the following sections.

### 2.1. Experimental Setup

The experimental setup for assessing the performance of the ABC algorithms consists of ImageLab, which is an in-house image quality assessment (IQA) tool for endoscopy applications, test charts, and endoscopy systems.

#### 2.1.1. ImageLab

**Figure 2:**
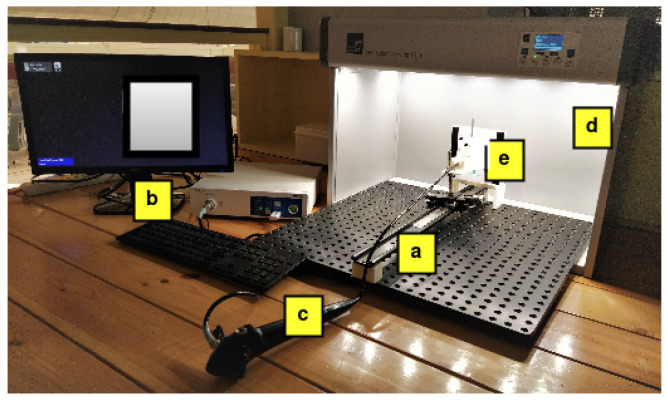
Experimental setup. (**a**) Endoscope railing to allow the adjustment of the distance of endoscope tip from the test chart. (**b**) Endoscopy system. (**c**) Endoscope mounted on the railing. (**d**) Illumination box with standard D65 light. (**e**) Test chart.

ImageLab consists of an experimental setup inside an illumination box providing standard D65 light and test charts, as shown in Figure 2. The test charts were placed on the holder at the end of the endoscopy railing, and the endoscope was held with the scope holder on the railing at the maximum working distance from the test chart. The railing enables the scope holder to be moved in the direction perpendicular to the viewing axis of the test chart so that the position of the scope position can be adjusted according to the point of desired focus on the test chart. The setup is described in detail in the previous works [18,20,21]. The assessment was carried out in a dark room to avoid any influence of stray light on the assessment results. Since the experiments were focused on evaluating the endoscope’s illumination, the illumination box was not utilised in the current study.

#### 2.1.2. Test Charts

**Figure 3:**
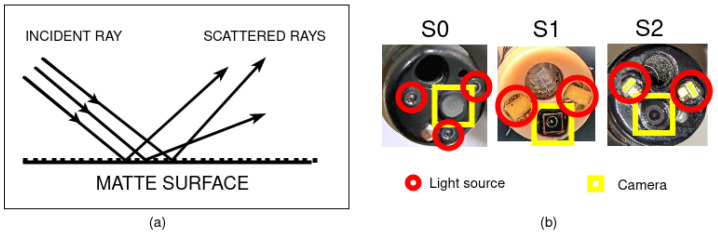
(**a**) Scattering on a matte surface. (**b**) Scope tips of the endoscope systems. S0 has 3 sets of light sources around its camera, while S1 and S2 have only 2 sets of light sources adjacent to their cameras.

Seven test charts—white, red, green, blue, cyan, magenta, and yellow—were printed using a 4 × 6 inches digital printer at 300 dots per inch (dpi). A matte photo print was employed to minimise the shine of the images captured and refract incident light from the scope light source. The print material is chosen based on its ability to scatter light in multiple directions rather than reflecting it off the surface due to the tiny indentations of the matte finish [22], as shown in Figure 3.

#### 2.1.3. Endoscopy Systems

The endoscopy systems used for this study were:

**S0**—A commercially available Olympus CV-190 gastroscopy system;

**S1**—An in-house development bronchoscopy system;

**S2**—An in-house development gastroscopy system.

S0 has a Xenon short-arc lamp connected to the light source unit CLV-190 with a servo-diaphragm technique for ABC [23]. The development systems S1 and S2 utilise LEDs as light sources controlled using proportional-integral (PI) controllers. S1 operates on a single PI controller for the LED light intensity control as its camera exposure is always set to maximum. S2 has two PI controllers, one each for LED light intensity and camera exposure. The control parameters of the ABC algorithms of the development systems were set to ensure optimal performances of respective systems. The scope tips of the three systems are shown in Figure 3b. Their image sizes are 1280 × 960, 800 × 800, and 1440 × 1080, respectively, and render video at 50, 60, and 60 frames per second (fps).

### 2.2. Experiments

Initially, the endoscopy systems were white-balanced with their respective light sources for producing true colours [24]. The ABC of the systems was enabled by default, and each endoscope was fitted at its respective maximum working distance to sequentially observe the center of the test charts. The PI controller parameters were tweaked in S1 and S2 to analyse their impact on the corresponding illumination and response changes. Three emulations were carried out:

**E0**—Optimal controller response for S0, S1, and S2;

**E1**—Controller response slower than the optimal response for S1 and S2;

**E2**—Controller response faster than the optimal response for S1 and S2.

The experiment was started by turning off the scope lights, and the camera was closed manually with a sheet to prevent any adjustments through light intensity or camera exposure controls. Subsequently, the camera sheet was opened, and the light was activated simultaneously to record a video of the ABC response from least brightness (dark) to optimal brightness using a frame grabber. Frames were extracted from the video, and individual image frames were utilised as input for the algorithms developed for the assessments in MATLAB R2020 [18,20,21].

#### 2.2.1. Assessment 1 (A1)—Image Brightness and Homogeneity of Brightness

**Figure 4:**
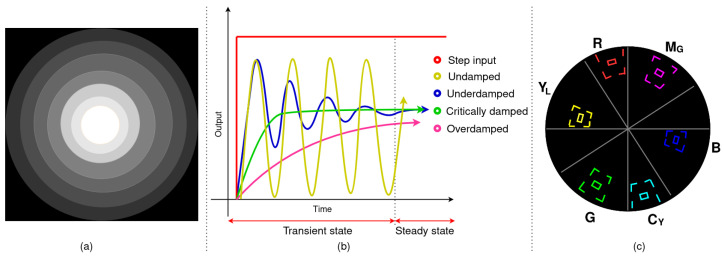
(**a**) Illumination profile depicted by clustering the bins of the histogram of the observed image frame. (**b**) Transient response of a second order system with different damping ratios when a step input is given. (**c**) Vectorscope designed with the NTSC graticule as reference.

In this assessment, images were captured observing the white test chart, and the histogram was plotted to provide a global description of each image [25]. From the histogram, the illumination profile or pattern formed was depicted by clustering the intensity values into eight bins, as shown in Figure 4a.

#### 2.2.2. Assessment 2 (A2)—Controller Response and Response Time

For any dynamic system in real-time, the output controller response follows a transient response to finally settle at the desired steady state [26] by decaying the oscillations, which, in other words, is called damping [27]. Consider a second-order system with an open-loop transfer function [28]

(1) $$G\left(s\right)=\frac{\omega_{n^{2}}}{s(2\zeta \omega_{n}s+\omega_{n^{2}})}$$

The Laplace of the transfer function of a closed-loop system for output signal c(t) and input step signal r(t) with unity negative feedback is obtained from Equation (1).

(2) $$\frac{C(s)}{R(s)}=\frac{G(s)}{1+G(s)}=\frac{\omega_{n^{2}}}{s^{2}+2\zeta \omega_{n}s+\omega_{n^{2}}}$$

where $\omega_{n}$ is the natural frequency at which the system oscillates and ζ is the damping ratio that characterises the frequency of the oscillations.

The characteristic equation is obtained from the denominator of Equation (2)

(3) $$s^{2}+2\zeta \omega_{n}s+\omega_{n^{2}}=0$$

The roots of the characteristic equation are determined by the damping ratio ζ

ζ = 0    : undamped system;

0 < ζ < 1 : underdamped system;

ζ = 1    : critically damped system;

ζ > 1    : overdamped system.

Figure 4b highlights the effect of damping on a second-order system. In this assessment, the illumination response of the ABC algorithm at the desired brightness level through damping is analysed. The number of pixels in the brightest intensity band was tracked across the frames upon illumination for each system. The response curve was plotted, and if it was an underdamped response, the damping ratio was determined using logarithmic decrement δ.

(4) $$\zeta =\frac{\delta }{\sqrt{\delta^{2}+4\pi^{2}}};\;\delta =ln\frac{x_{0}}{x_{1}}$$

and *x*_0_ and *x*_1_ are the amplitudes (number of pixels) of successive peaks [28].

The response time *t*_r_ in seconds was calculated using Equation (5) by dividing the number of frames taken for the controller to settle at the desired brightness by the frame rate of the system.

(5) $$t_{r}=\frac{Number\;of\;frames\;taken\;to\;settle}{Frame\;rate\;of\;the\;system}$$

#### 2.2.3. Assessment 3 (A3)—Colour Rendition

Colour rendition is the ability of a light source to render the colours of the scene accurately [24]. In this assessment, the graticule of the NTSC (National Television System Committee) was taken as the reference to design a vectorscope (Figure 4c) to represent the colour information in the observed images [29]. The hue and saturation of pixels vary axially and radially on the vectorscope, respectively [30]. There are six target shapes with smaller shapes representing the ideal levels of red (R), green (G), blue (B) (referred to as RGB), cyan (C_Y_), magenta (M_G_), and yellow (Y_L_) (referred to as CMY). The pixel information from the images of the white and colour test charts captured was plotted on the vectorscope to assess the colour rendition capabilities with respect to the illumination.

The results of the experiments conducted on the three endoscopy systems for all of the emulations are presented in the next section.

## 3. Results

The results of the performance of the ABC algorithms of the endoscopy systems S0, S1, and S2 for the three emulations E0, E1, and E2 are presented below.

### 3.1. A1—Image Brightness and Homogeneity of Brightness

**Figure 5:**
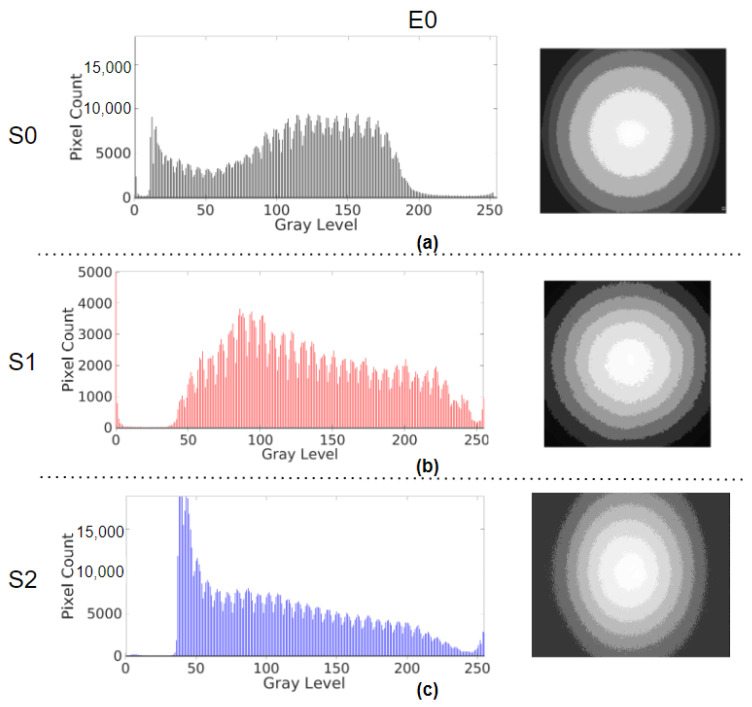
Histogram of the frame after the response has settled and illumination profile of E0 in (**a**) S0, (**b**) S1, and (**c**) S2.

**Figure 6:**
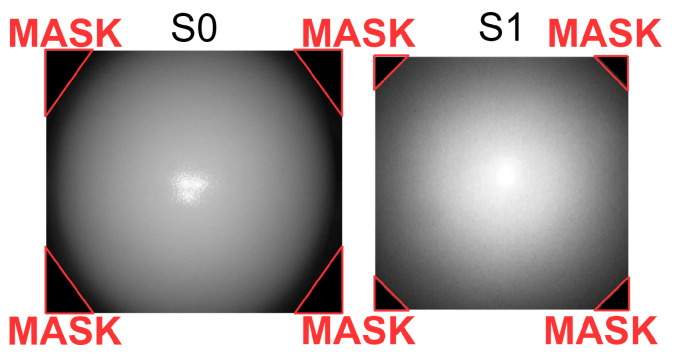
Image masks of S0 and S1, which are present in all of the endoscopy image frames.

**Figure 7:**
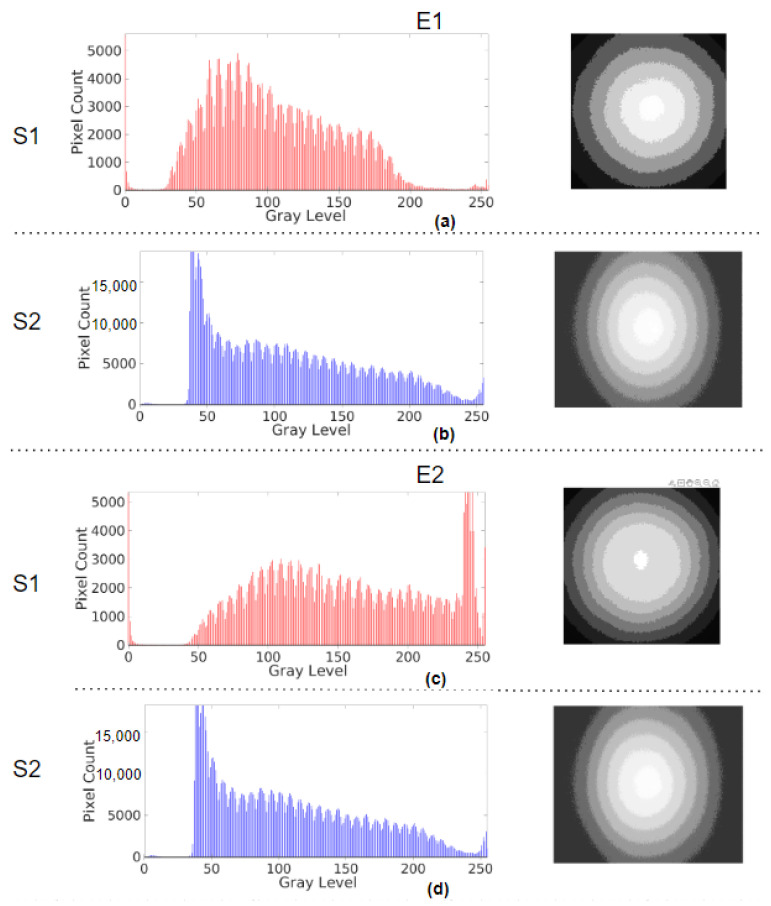
Histogram of the frame after the response has settled and illumination profile of E1 in (**a**) S1, (**b**) S2 and E2, (**c**) S1, and (**d**) S2.

The image brightness and illumination profiles of the systems for all of the emulation cases are summarised in Table 1. The histograms and the corresponding illumination profiles, after the control response of the ABC algorithm has settled or reached a steady state, are shown in Figure 5. The peaks at zero intensity level in histograms were outliers in S0 and S1 as they were from their image masks (Figure 6). The number of pixels above the intensity value of 240 was due to glare from the light source of the system light source and hence not considered in determining the range of illumination in the histograms. Only the intensity values with a number of pixels above 1000 were considered in the range. Figure 5a–c highlight the E0 response of S0, S1, and S2 having intensity ranges from 11 to 195, 46 to 231, and 35 to 231, respectively. The noise levels between 10 and 11 in S0, 15 and 22 in S1, and 37 and 47 in S2 were observed in their dark frames, which could have caused the corresponding peaks in their histograms. S1 was unaffected by the noise level, but the minimum intensities of S0 and S2 were considered 12 and 48. The ranges were 183, 185, and 184, which were calculated from the difference between their respective maximum and minimum intensities. Figure 7a,b are the histograms of the images for the E1 on S1 and S2. For S1, the range is between 36 and 187, and the distribution was shifted and skewed towards the left as compared to the E0 response. For S2, the range and distribution remain the same as observed in E0. Figure 7c,d show the E2 response of S1 and S2, respectively. S1 settles with a glare as indicated by the peak between 240 and 250, whereas S2 again settles with a distribution similar to E0.

In the illumination profile, the intensity bands denote the brightness range of the pixels in the ROI of the image, and each range has thirty-two intensity values. In E0, seven, seven, and eight bands were observed in the frames in S0, S1, and S2 when the illumination control settled. The brightest band was the glare at the center, and from there, the intensity decreased towards the periphery. In the E1 response, S1 had only six bands covering the image frame, wider than the bands observed in the E0 case, and S2 similarly had eight bands as its E0 case. In the E2 response, S1 had the same seven bands but the widths of each band varied compared to the E0 response, and S2 again had eight bands, as seen in the E0 case.

### 3.2. A2—Controller Response and Response Time

**Figure 8:**
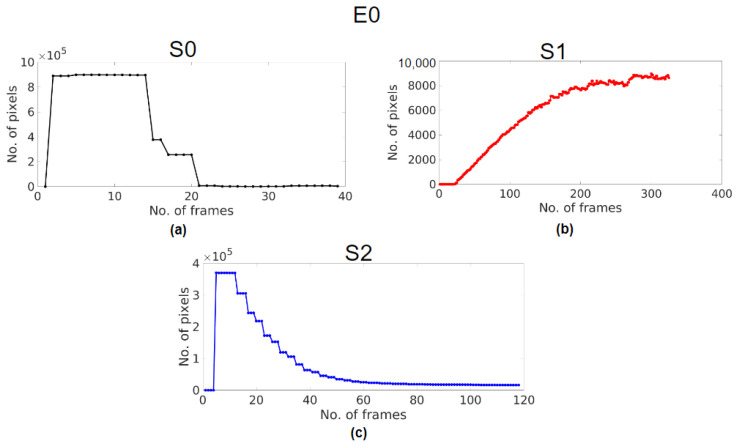
Response curve in E0 for (**a**) S0, (**b**) S1, and (**c**) S2.

**Figure 9:**
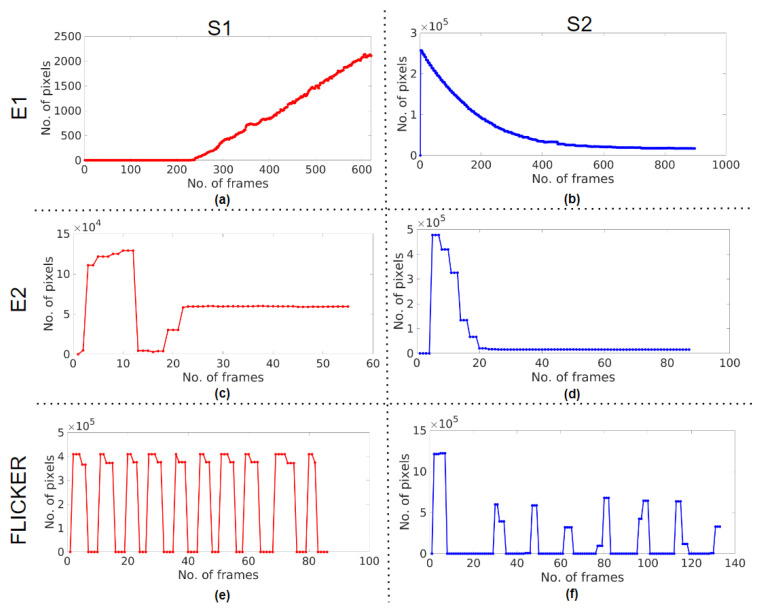
Response curve in E1 for (**a**) S1 and (**b**) S2, in E2 for (**c**) S1 and (**d**) S2, and when flickers occur in E2 for (**e**) S1 (**f**) S2.

Figure 8 and Figure 9 are the controller response curves plotted from the brightest intensity bands obtained from the illumination profiles in A1. Table 2 summarises the response characteristics of all of the cases for all of the systems observed. The damping ratio ζ of each response was calculated using Equation (4). ζ of S0 in case E0, and S2 in cases E0, E1, and E2, was calculated from the peaks of their respective responses. The damping ratios of S0 and S2 in E0 were 0.597 and 0.394. S1 does not have peaks, which could be because of excessive damping, and so ζ could not be calculated using Equation (4). Since the E1 case shows a response slower than the E0 case, the S1 response seems to experience more damping, leading to no peaks, while S2 has a ζ of 0.333. In the E2 case, the damping seems to have decreased for the S1 response as it has both positive and negative peaks with a ζ of 0.519, and S2 has a ζ of 0.471.

The response time was calculated by observing the number of frames taken to settle at a steady state using Equation (5). The response of S0 shows that the number of frames taken to settle was 24, and so its response time was 0.400 s. In the E0 case for S1, the response seemed to properly settle only after 300 frames, and so the response time was greater than 5 s, and for S2, the response in the E0 took 70 frames, which meant that the response time was 1.167 s. In the E1 case, both S1 and S2 took over 600 frames to settle, which was over 10 s. In the E2 case, S1 took 22 frames and S2 took 23 frames; the respective times of response calculated were 0.367 s and 0.383 s.

In real-time, the controller response must be stable even when the scope tips are placed at their minimum working distances from the test chart. The response times in the E2 case for S1 and S2 were shorter than those of S0 in E0; Figure 9e,f show the controller responses, which never attain a steady state but rather lead to the flickering of the light. Though some damping was observed in S2 initially, it was negligible to calculate and was almost 0.

### 3.3. A3—Colour Rendition

**Figure 10:**
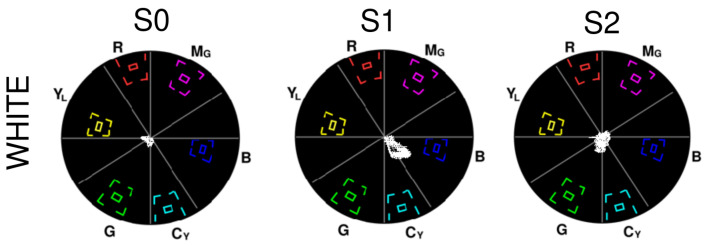
Rendition of white on vectorscope by S0, S1, and S2.

**Figure 11:**
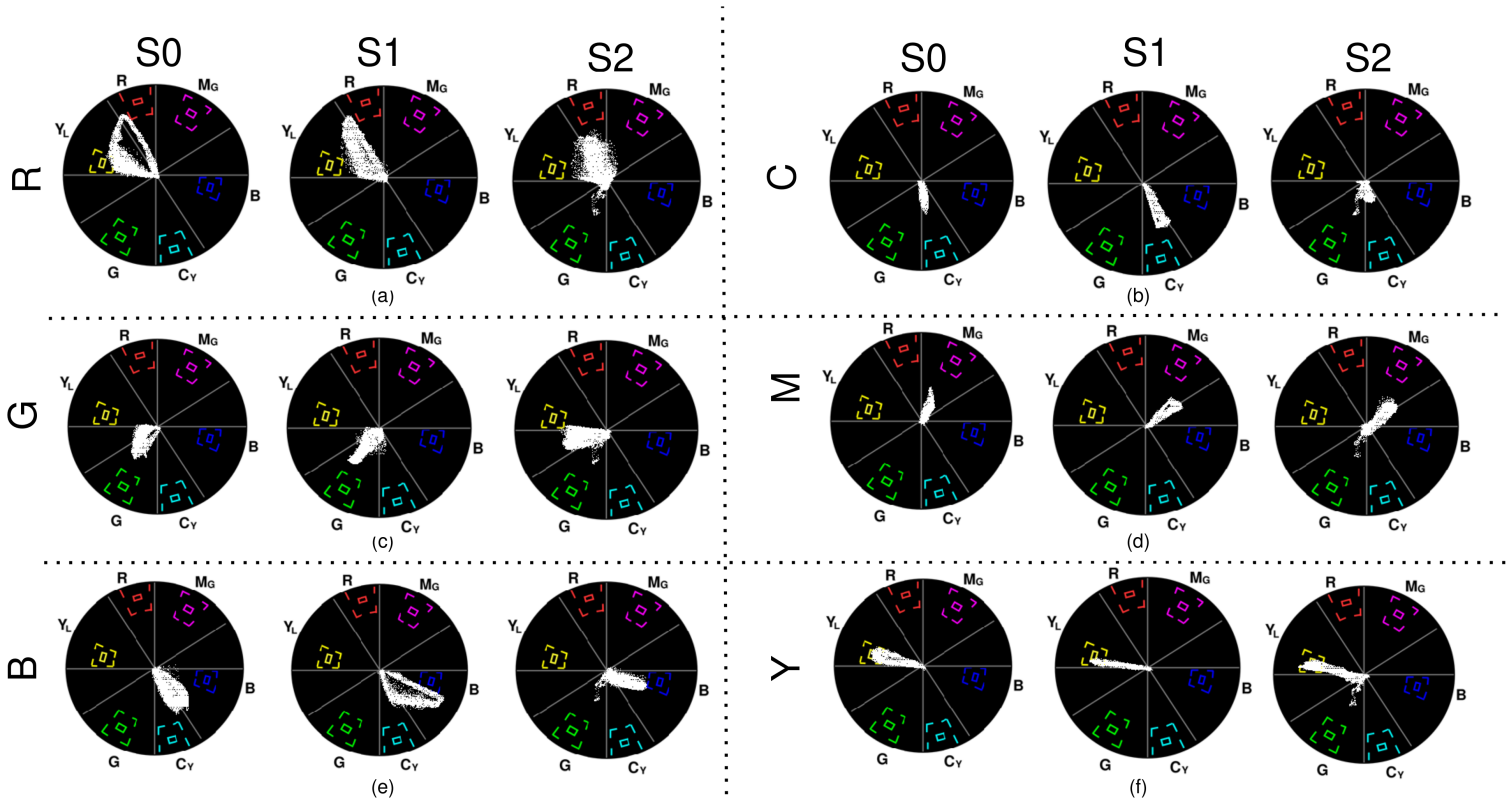
Rendition on the vectorscope. (**a**) Red (R) by S0, S1, and S2; (**b**) cyan (C) by S0, S1, and S2; (**c**) green (G) by S0, S1, and S2; (**d**) magenta (M) by S0, S1, and S2; (**e**) blue (B) by S0, S1, and S2; and (**f**) yellow (R) by S0, S1, and S2.

Since the colour rendition for the entire frame was completed, a significant number of pixels in the center of the vectorscope could be seen due to glare. Figure 10 and Figure 11 show the colour rendition for white, RGB, and CMY in all of the systems that detail the hue and saturation of each colour rendition. It is to be noted that S1 has some pixels constantly rendered as blue and S2 has some pixels in green, which could be chroma noise.

- **White:** S0 contains some amount of blue and green, S1 is skewed towards blue and cyan, and S2 contains some green.
- **Red:** S0 has most of the pixels within the yellow and orange phase and the remaining pixels in red. The maximum saturation obtained in the red phase is low. S1 renders red as orange, and the pixels in the red phase are very low in saturation. The red in S2 is spread across red, yellow, orange, and even magenta, with low saturation.
- **Green:** S0 renders green and deep green with some yellow at low saturation. S1 renders in deep green and yellow with better saturation than that observed in S0. Rendition in S2 is similar to S0 but with higher saturation. None of the outputs are close to the maximum chroma value of green.
- **Blue:** S0 has pixels in both blue and cyan, and the maximum blue saturation is close to the chroma gain of blue. Though S1 has some cyan too, its blue rendition is good, and many pixels fall within the bigger target for blue. S2 renders blue well but with less saturation.
- **Cyan:** S0 has pixels that fall between cyan and green with low saturation. S1 renders cyan well at better saturation than S0 but is still low compared to the cyan target. S2’s cyan is whiter due to very low saturation.
- **Magenta:** S0 renders magenta well with low saturation. S1 and S2 almost render magenta the same at similar saturation but have a few pixels spread in purple.
- **Yellow:** Yellow was rendered properly by all three systems, with many pixels achieving the required saturation by reaching the bigger targets. S2 has some pixels within the inner target too.

A detailed discussion of the observed results is discussed in the following section.

## 4. Discussion

This study evaluates the effectiveness of the automatic brightness control (ABC) algorithms of endoscopy systems by assessing image brightness and homogeneity, controller response and response time, and colour rendition. The emulations conducted in the study demonstrate the tradeoffs in ABC performance in terms of the image illumination, speed, and stability of the controller response. The transient responses of the ABC algorithms plotted illustrate the damping of the controller response, from which system stability is deduced, and also highlight cases of slow response and flickering. The results also show how the illumination profile affects the hue and saturation of the image, leading to colour shifts in the observed image. Additionally, the observations suggest the need to observe the illumination profile rather than just the histogram for assessing image illumination. Furthermore, the interdependency among the assessments provides a holistic explanation of overall image quality. This study highlights the need to properly design and optimise ABC algorithms to avoid tradeoffs in performance that can result in perceivable inconvenience during real-time endoscopy examinations, which could hinder diagnosis and therapy.

**Figure 12:**
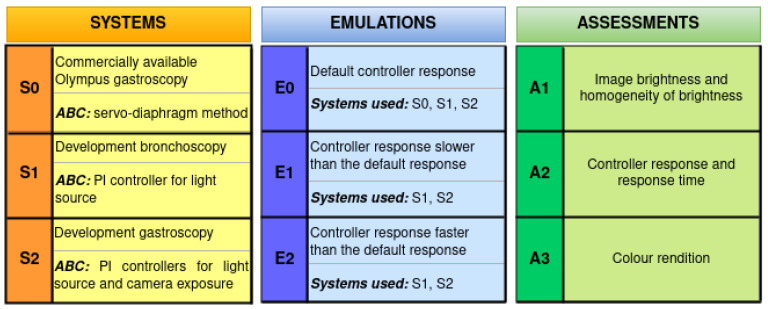
Lists of the endoscopy systems (S0, S1, and S2), the emulations (E0, E1, and E2), and the assessments (A1, A2, and A3) employed in this experimental study.

Figure 12 consists of the lists of systems, emulations, and assessments used in this experimental study. Some observations in the results of all of the assessment techniques showed prevalent features in the images of the endoscopy systems. In A1, the observed histograms had abrupt peaks (Figure 5 and Figure 7) denoting black image masks (intensity zero), noise level, and glare. Additionally, since the scope axis is perpendicular to the test charts in this study, the observed illumination profile is concentric in nature; the brightest band is in the center of the image, and it gets darker towards the periphery (vignetting). This agrees with the point that the illumination profile aids in the design of light sources suitable for application and placement near the camera sensor [18,31,32,33,34].

In A2 (Figure 8 and Figure 9), the controller response from the ABC algorithm of the endoscopy systems mimics a second-order transient response trying to settle at an optimal illumination level. In this experiment, when the light is turned on, there is a sudden influx of illumination, and the system considers this input signal as a disturbance. Being a closed-loop system with ABC control, the system behaves like a high pass filter to control the resultant brightness following a disturbance-rejection response with an exponentially decaying curve [28].

In A3, the inner targets on each colour of the vectorscope (Figure 10 and Figure 11) will ideally contain a dot representing the colour with constant hue and saturation throughout the ROI or image observed [30]. However, instead of a dot, due to the influence of light that is shown by the illumination bands or the presence of noise in the signal, the pixels are fuzzier or spread out radially and axially on the vectorscope, which causes spectral shifts of the observed colour.

A closer look into the results of emulations (E0, E1, and E2) revealed the specific behaviour of the ABC algorithms of each system. In the E0 case, from the observed histogram in Table 1 and Figure 5, though the intensity range (calculated from the maximum and minimum intensities) of S0 (Figure 5a) is smaller than that of S1 (Figure 5b) and S2 (Figure 5c), the S0 image seems to be brighter. Upon observing their illumination profiles, the intensity bands of S0 are larger at the center and smaller towards the periphery, as opposed to equally spaced bands of S1 and S2. This means that there is a high concentration of illumination at the center of the image in S0, which could be due to the way its light source is designed, making it effectively achieve brighter images with less illumination. From this case, it is to be noted that observing only the histogram data could be misleading, and the homogeneity of the brightness helps in further understanding the image brightness.

Upon observing the controller responses in Table 2, the controller responses of S0 (Figure 8a) and S2 (Figure 8c) are underdamped with damping ratios ζ less than 1, while that of S1 (Figure 8b) is overdamped with a ζ greater than 1. Practically, an underdamped system is preferred and more feasible in real-world applications [35], and so the responses of S0 and S2 are preferred as opposed to S1. S0 has the shortest response time of all, S1 does not settle at all, and S2 takes more than 1 s to settle. However, the response time is preferred to be less than one second for endoscopy applications so that there is no perceivable inconvenience during a real-time examination [13] and so the performance of S0 is preferred over S2.

The E2 case is emulated to improve the controller response characteristics and reduce the response time for S1 and S2 [36,37]. S1 (Figure 9c) achieved an underdamped response with ζ and a response time close to that of S0 in E0, as shown in Table 2. However, it is to be noted that the response consists of a negative peak that, in real-time, is a flicker that could be unpleasant to the eyes of the user [38]. This shows that the controller response could be an indicator of the system stability too. After the response settles, the histogram and the illumination profile of the frame show that the image has glare, which could mean that the response has settled with an offset as shown in Figure 7c. For S2, though the response is still underdamped, the response time is close to that of S0 in E0, as shown in Figure 9d. However, S1 and S2 at their minimum working distance result in flickers, as shown in Figure 9e,f (characterised by oscillations in the response with the same frequency), which is much like an undamped system (ζ = 0) that never settles and so is not preferred [36,37]. Thus, the ABC algorithms of S1 and S2 did not perform as desired in the E2 case.

Thus, the speed and stability of a system depend on its controller response, which is heavily influenced by damping [36,37]. Ideally, a controller response with ζ of 0.707 [39] reaches a steady state rapidly. Though efforts to achieve the ideal ζ could be made to attain a steady state with a minimum response time, the stability of the system must be maintained. Table 2 shows that S0 in E0 and S1 in E2 have ζ closest to 0.707, but only the response of S0 reaches its steady state with stability in its range of working distance. S1 results in an unstable response with flickers, and so necessary corrections in the ABC algorithm must be made to achieve a stable control response.

The E1 case is examined to highlight the characteristics of the slow response of the systems. For S1, the left-shifted and skewed histogram in E1 (Figure 7a) could mean that the response of the control algorithm has not completely settled, as shown in Figure 9a, thereby resulting in insufficient illumination, as indicated by its maximum intensity. Though the intensity bands are almost equally spaced, Figure 7a shows that there are only six bands as the overdamped response is yet to settle, resulting in a sluggish response. For S2, the histogram (Figure 7b) is the same as in E0 and E2, with an underdamped response, but also has a sluggish response as seen in Figure 9b. Thus, in this case, S1 and S2 are not suitable for endoscopy applications [5,13].

Colour rendition is observed after the illumination achieves a steady state with good image brightness, as shown in Figure 10 and Figure 11. The illumination of S0 shifts a few pixels from its respective hues while observing RGB and CMY, and most of the pixels are clustered near the center of the vectorscope. Such behaviour could be due to the concentration of the light emitted from the light source on the image as observed in its illumination profile. Only in S1 and S2, a constant spread of pixels indicated by the outliers on the vectorscope in blue and red, respectively, is observed, which could be due to an undesirable reflection of light. The vectorscope results of S2 show lesser colour shifts and better saturation, compared to S0, due to its uniform illumination profile. For S1, since it has a uniform illumination profile with an overdamped response where the illumination is less exposed, the output of the vectorscopes had better saturation than S0 and S2, with fewer pixels towards the center. Thus, S1 has better colour rendition capabilities than S0 and S2.

Thus, a comprehensive assessment of the ABC algorithms in the three systems considering the interdependency among the three assessment methods gave the following critical insights. The ABC algorithm of the commercial endoscopy system S0 performed better than the in-house developed systems S1 and S2 by achieving good image brightness steadily and rapidly. Amongst S1 and S2, the performance of S2 being better than S1 could be due to the implementation of two control algorithms, each for camera exposure and light source, as opposed to only one algorithm for controlling light source in S1. However, S2 still needs better tuning of the control parameters to achieve the performance of S0. S1 renders colours better than S0 and S2 at lower light intensities, and S2 renders colours satisfactorily with good image brightness as compared to both S0 and S1.

Based on the discussion of the results, the proposed and related works could be compared.

- **Objective Assessment:** Though subjective assessment from experts could be useful as performed by McCallum et al. [11], it is unreliable unless there is a substantial amount of feedback, which makes it a time-consuming process. The proposed methodologies are objective assessment approaches that provide quantitative (A1 and A2) and qualitative (A3) assessments of the image data.
- **Comprehensive Assessment:** Shreshtha et al. [12] assess only image brightness and its homogeneity, and Cavallotti et al. [17] assess only the controller response. Sousa et al. [13] do not consider the assessment of colour rendition. To assess the overall performance of ABC, all of the assessment methodologies considered in the proposed work need to be considered to achieve homogeneous brightness rapidly and steadily without any colour shift.
- **Blind Assessment:** Cavallotti et al. [17] assess controller response based on the power consumption of the system which, would require access to the endoscopy hardware. Sousa et al. [13] also calculate response time from their endoscopy hardware. The results of the proposed methodologies could be carried out with only the endoscopy image data.

Thus, the proposed work is suitable for assessing the overall ABC performance of any endoscopy system.

## 5. Conclusions

In conclusion, this work presented a comprehensive assessment of the effectiveness of automatic brightness control (ABC) algorithms in endoscopy systems, taking into account the interdependency among the image brightness and the homogeneity of brightness, the controller response and the response time, and the colour rendition. Our findings indicate that the interdependency among the proposed methods can establish tradeoffs in the overall performance of the ABC algorithms better than single-parameter approaches. The study establishes that comprehensive assessments using the proposed methods can contribute to designing new ABC algorithms and optimising already implemented ones for efficient performance in endoscopy systems.

## 6. Limitations and Future Work

The limitation of this work is that the experiments were conducted on only three systems with different ABC algorithms. Moreover, the subjective opinion of experts on the ABC algorithms, which could give insights into their preferences, was not taken into consideration as it was beyond the scope of this paper. However, as a part of future work, multiple systems with different endoscope tips and ABC algorithms could be considered for study to observe the effectiveness of the assessment methodologies. Additionally, a score-based technique could be implemented based on the feedback of experts, which would accelerate the assessment process and provide confidence in the results.

## Figures and Tables

**Table 1:** Results of (1) histograms: minimum intensity (I_min_) after rejecting the noise levels (10–11 in S0, 15–22 in S1, and 37–47 in S2); maximum intensity (I_max_); and range of histogram (RANGE), which is the difference between I_max_ and I_min_, and (2) number of bands in the illumination profiles (IP) for the systems in all three emulation cases.

*Emulations*	*Systems*	I_min_	I_max_	RANGE (I_max_ − I_min_)	IP
	**S0**	12	195	183	7
**E0**	**S1**	46	231	185	7
	**S2**	48	231	184	8
**E1**	**S1**	36	231	195	6
	**S2**	48	231	184	8
**E2**	**S1**	46	250	204	7
	**S2**	48	231	184	8

**Table 2:** Illumination response summarising damping ratio and response time for the systems under observed cases. (“X” denotes that the emulations were not applicable to the system, and “-” denotes that the calculations were not possible for the undamped systems).

*System/Emulations *	*Damping Ratio (No Unit) *			*Response Time * (s)		
	E0	E1	E2	E0	E1	E2
**S0**	0.597	X	X	0.400	X	X
**S1**	-	-	0.519	>5	>10	0.367
**S2**	0.394	0.333	0.471	1.167	>10	0.383

## Data Availability

Videos and extracted image frames of S0, S1, and S2: https://drive.google.com/drive/folders/1KZUReUrAWDz-w-L-bXwlWtARscdFjYvH?usp=sharing, accessed on 8 April 2023.
